# Women elite Italian football players' perceptions on gender equality and dual career opportunities

**DOI:** 10.3389/fspor.2025.1508147

**Published:** 2025-05-06

**Authors:** Flavia Guidotti, Simone Ciaccioni, Sabrina Demarie, Piersanto Colombo, Cristina López De Subijana, Elvira Padua, Laura Capranica

**Affiliations:** ^1^ Department of Human Sciences and Promotion of the Quality of Life, San Raffaele Roma Open University, Rome, Italy; ^2^ Department of Movement, Human and Health Sciences, University of Rome “Foro Italico”, Rome, Italy; ^3^ Department of Wellbeing, Nutrition and Sport, Faculty of Human Sciences, Education and Sport, Pegaso Telematic University, Naples, Italy; ^4^ Social Sciences Applied to Sport, Physical Activity and Leisure Department, Universidad Politécnica de Madrid, Madrid, Spain

**Keywords:** gender equality, football, dual career, questionnaire, thematic analysis

## Abstract

**Introduction:**

The aim of the present study was to explore dual career experiences of elite women Italian football players in light of potential gender inequalities that might have affected their sporting and academic career paths.

**Methods:**

A 25-items semi-structured questionnaire was administered to 22 elite Italian football players (age: 25.8 ± 4.3 years).

**Results:**

The mixed-method thematic analysis highlighted participants' difficulties in combining sport and education (e.g., lack of time, lack of dual career opportunities), reporting a higher support received from the sporting context (e.g., training absences and schedule adaptation) rather than from the educational institutions (e.g., lack of flexibility for class absences and for exams/evaluation schedule). Seven participants only benefited from the recognition of the “student-athlete status” and the formal access to a dual career program, mainly due to gender differences in the recognition of eligible competitive levels and past gender equality policies. Furthermore, the gender pay gap in professional football was perceived as a crucial factor in determining women's major interest in academics and educational achievements over their football performance development.

**Discussion:**

The present findings reflect previous literature in this field, with the football career perceived as unsecure and precarious, even more for women in such male-dominated industry, urging them to prioritize their education. Although the recent professional recognition was positively perceived, the present study highlighted the need for further research in this area. Furthermore, the translation of evidence-based information into further policy implementation towards gender mainstreaming and equal dual career paths in football should be envisioned.

## 1 Introduction

Sustainability in sports represents both a great challenge and an opportunity (1), with gender mainstreaming and quality education representing key factors to pursue strategic objectives of sustainable development (2). In this framework, gender inequalities and dual career (e.g., the pursuit of both elite athletic performance and academic or vocational development, allowing individuals to balance their sporting demands with education and/or professional career aspirations) issues still permeating several sporting contexts urge policy interventions, recommendations, the dissemination of good practices, and concrete actions for the promotion and monitoring of both gender mainstreaming and the safeguard of elite sportspersons' rights to education (3–7). It is well documented that a considerable gender inequity in sports still persists (6–8), with prevalent discriminatory practices affecting several sports disciplines and professional profiles (e.g., professional athletes, coaches, sport mangers/directors, referees). Furthermore, being a sports career a dynamic, multidimensional, multilevel, and multifactorial process (9), the progressive increase of athletic demands, dual career requirements, and socio-cultural and life aspects strongly impact career transitions (e.g., from the youth talented stage till retirement and post-athletic career) of elite sportspersons (9–20). Thus, sport and academic support, facilitating external socio-cultural environments, financial resources, and services are key in determining athletes' career trajectories, also in relation to their gender (9, 11, 13, 21–24).

Globally, football represents the most popular sport worldwide (25, 26), accounting for the highest share of both participants and fans (e.g., around 270 million and 4 billion of people, respectively). Since its establishment in the 19th century in England, football was mostly played by boys and men, with several antecedent forms of play (e.g., wild folk games) where males fought against their opponents while striving to score a goal. Since its early developmental stages, football was included in English education programs for young boys to learn self-control and rule compliance, and was fully embraced by the working classes with matches attracting massive masculine audiences (27–29). Conversely, women's football struggled to gain popularity in terms of both public attention and participation (26, 30–33). Even today, football represents a social ground for the expression and reinforcement of hegemonic masculinity, legitimating discriminatory hierarchical relations between genders (27–29). Being strength, speed, muscularity, and competitiveness the dominant characteristics of the game, traditionally men were given more opportunities and better conditions to pursue an elite level career with respect to their women counterparts, with financial gaps and lack of professional recognition having affected female players' career trajectories till recent times (30, 31, 34). Furthermore, females were found to prioritize education and work outside the elite football environment, as confirmed by several studies (27, 35–39) suggesting a higher drop-out rate due to unfair football conditions with respect to their male counterparts.

Gender mainstreaming in sport has become a crucial priority at European level (5). The establishment of a dedicated “High Level Group on Gender Equality” to provide recommendations and an action plan to increase the share of women and girls in sport participation, coaching and officiating, and leadership positions reflects the European Commission's commitment in this area (5). Strategic areas of intervention pertain also the promotion of gender equality in social and economic aspects of sport, equity in media coverage, and the fight against sports-related gender-based violence. In general, international recommendations are adopted differently within national contexts and translated into national strategies for implementation. Worldwide, given the variety of national contexts in terms of general and/or specific sports policies, as well as conditions for elite sportspersons recognition and working welfare, national action plans are crucial to determine a tangible impact. Hence, governmental and sports organizations formulate targeted policies, support projects, and sustain concrete interventions to promote gender mainstreaming at different levels (e.g., grassroot sport participation, support services for elite athletes, welfare and equality for sporting professionals). In top-level football, the establishment and strengthening of national and international women's competitions, the recognition and management of women's football by major umbrella sports organizations (e.g., the Union of European Football Associations, UEFA; and Federation Internationale de Football Association, FIFA), and the inclusion of women's football in the Olympics represent milestones towards gender mainstreaming in this area (32). In particular, the major football governing body FIFA is strongly committed in reducing the gender gap launching the FIFA's Women's Football Strategy, sustaining the professional recognition of women players (40), and including gender equality in its strategic plan towards a global game (41). With the women's football movement rapidly evolving, national football federations worldwide complied with FIFA's strategic action plan and objectives by taking direct action and establishing their own national strategy towards more women and girls in football at all levels. In the past decade, various reconfigurations within the different national contexts have been observed (40). An example is the recent restructure of English football in the sport season 2018/19, requiring all clubs participating in the women's first league to move to the full-time status. This transition should be considered a milestone for women elite players as professionals, with football becoming a tangible career opportunity (40). In parallel, national teams' successes at international level are also crucial for attracting sponsorships and investments to increase the overall economic and cultural impact of the sport within national contexts, as well as a measure of women empowerment (34).

Since its early appearance in the 30s, the development of Italian women's football has been characterized by a slow and progressive growth, leading to the establishment of the first National (informal) Serie A championship in 1968. However, in 1986 only the Women's Seria A became an official competition. Since then, only 19.000 female players were registered at the Federazione Italian Giuoco Calcio (FIGC) in 2008–2009, whereas in 2019–20 a 65% increase was recorded, exceeding 31.400 women members. Hence, FIGC took ownership of the management and organization of top-level national competitions (e.g., Serie A, Serie B, Primavera, Coppa Italia, and Supercoppa) (42). In parallel, also the television coverage, social media engagement, and the overall economic impact of women's football have increased, leading to the adoption of a Strategy 2020–2025 (43) and the establishment of the professional status for female football players in 2022 (42). At present, Italian men's and women's football occupies the 10th and 15th rank position in their respective world ranking, substantiating the strong tradition and cultural engagement of the sport at national level (44). Furthermore, given the increasing relevance of Italian women's football at large since the professional status of female players has been recognized, regulatory frameworks for the protection their interests have been also implemented (45).

Regarding dual career, no formal policy is in place in Italy, with a legislative reference only for the protection of high school student-athletes' right to education (46). To meet the needs of youth talented male football players, the top-level club academies complement their football training and competition programs with residency, educational support, tutoring, and counselling services, and establish agreements with local educational institutions. Conversely, similar dual career opportunities are limited for female players. Despite the recent publication of the national dual career guidelines at higher education level and the inter-institutional agreement protocol on dual career (47) (e.g., including institutional efforts to promote dual career of elite athletes, identification of tools and resources to implement dual career programs, enhance the dialogue with elite sporting bodies and military sport groups, provide visibility to elite student-athletes enrolled in a dual career program, enlarging own networks of dual career stakeholders), a limited number of Italian universities formally adopt internal rules and *ad hoc* dual career programs for athletes, and apply different eligibility criteria (e.g., competition level, type of sport, training and competition volume, other criteria) to recognize the “student-athlete status” and/or the “elite sportsperson” status necessary to benefit of dual career-related flexibility and services (47–49). Furthermore, the association of Italian Universities interested in sport (e.g., Unisport Italia) provided a platform to inform athletes on dual career opportunities adopted by some higher educational institutions. Thus, before the professional women's football recognition in 2022, unequal dual career status recognition and opportunities might have occurred for Italian male and female players.

Over the past two decades, a growing scholars' interest regarding women's football mainly focused on its historical development and inequalities affecting female players' career paths (26–28, 30–33, 40), highlighting a substantial gendered pay gap despite comparable skills and dedication to the sport (50). To date, little empirical research has been conducted on the working status and conditions of women as elite professional players, mainly due to limited resources, lack of media coverage, and lower sponsorship investments and engagement (40, 51–53). Research on men's football career pathways highlighted that a professional football career is of short-term nature, both in contract length and career duration (54), precarious, and lacking long-term security (55, 56). Career trajectories of women players and the impact of the full-time status on their career development have also been investigated. Interestingly, several authors questioned the derived benefits and the long-term sustainability of professional women's leagues, also in considering that women's football careers have been shaped on a masculine model in a male-dominated industry (40, 51, 52). Research highlighted also that the gendered patterns in remuneration of male and female players determined a normative dual career path in elite women footballers in the past (57). Whilst the “chase of sporting dreams” has been always ascribed to the masculine gender, women are culturally expected to focus on their academic career and to take their traditional family role (53, 58, 59).

Transitioning from amateur to professional sport involves increasing dedication to rigorous training, competing at higher levels, managing contracts and sponsorships, and often adapting to a more demanding lifestyle (60), which might include a parallel dual career path. Athletes pursuing dual careers need time management, resilience, and networking skills, alongside academic or vocational knowledge, and they should therefore receive support from sports federations, educational institutions, and specialized career advisors (61). To date, female players pursuing dual careers sometimes receive support such as tailored training schedules, educational guidance, and mentorship programs. Although still rare and scattered, these resources aim to help them balance elite athletic performance with academic or vocational commitments effectively (62). To note, the recent establishment of the full-time status in women's football and the derived increased earnings might determine different trends in career choices and a possible shift in the dual-career discourse in the future. In fact, as already showed in men's football (54, 63–67), a dual career path might become a “backup plan” also in women footballers out of higher financial resources during their peak-performance years. However, it might be also plausible that gendered pathways in career choices will continue to be prevalent.

Given the global significance of football and the relevance of women's professional football as an emerging research area and discourse (26), it would seem appropriate to further explore and understand the intersection between the recent professional status of women football and the female players' dual career perceptions, needs, and opportunities. Despite the recent efforts of the FIGC towards the promotion of gender mainstreaming in Italian football (42, 43) and the raising awareness regarding the dual career needs of elite sportspersons at national level over the past decade, there is a need to fill the existing knowledge gap regarding the experiences of female football players in transitioning from amateur to professional sport. Evidence-based information could be key to guide future policy implementation in this area. Being dual career a right of the sportsperson, equal student-athletes' recognition and dual career support would nurture a well-educated workforce equipped with relevant knowledge and skills needed for productive and fulfilling employment after the sport career. In this framework, in line with the 2030 Agenda for Sustainable Development (2) a full participation in football-related career development and dual career should be guaranteed by adequate policies and actions towards gender mainstreaming in this area.

In exploring the potential gender inequalities and dual career experiences of elite women Italian football players, the present study represents the first attempt to investigate elite Italian female football players' perceptions regarding their sports and dual career trajectories, before the professional recognition recently occurred. It was hypothesized that to pursue better career opportunities Italian female football players might have prioritized one career over the other, potentially putting at risk their athletic success and/or jeopardizing future professional career prospects out of sport, also in relation to a gender discrimination characterizing football over the past decades.

## 2 Methods

### 2.1 Study design

The present study was performed under the Erasmus + Sport Collaborative Partnership “Women—new leader's empowerment in sports and physical education industry—New Miracle” co-financed by the European Commission (Project number: 622391-EPP-1-2020-1-LTSPO-SCP), and approved by the Institutional Review Board of the University of Rome Foro Italico (CAR 156/2023, May 17th, 2023, Rome, Italy), ensuring ethical standards have been guaranteed towards qualitative excellence and scientific rigor (68, 69).

An elite career (e.g., playing at the highest national competition level) in football implies athletes' facing relevant time demands and efforts for sport training and competition, which might be overloaded by their academic commitment and other social life aspects. Not requiring additional efforts to participants' dense sport and academic schedules, a semi-structured electronic questionnaire was considered appropriate to elicit participants' perceptions and opinions in relation to the quality, challenges, problems, concerns, and needs of their dual career path, in light of potential experienced gender inequalities (68, 70). According to the literature (71), open-ended questions embedded in a semi-structured questionnaire do not limit respondents' answers to specific closed-ended items, but rather stimulate the reflection on the proposed themes and the free expression of perceptions and opinions through an empty text entry field. Thus, both quantitative and qualitative thematic analyses have been considered to develop a contextualized understanding of the sport and academic career trajectories of the participants. In this framework, to highlight possible intersecting inequalities between dual career and gender equality in Italian women players, the following research themes were deemed crucial: (i) the management of the dual career path in Italian women's football; (ii) the perceived difficulties and challenges in pursuing a dual career, also in relation to potential gender inequalities; (iii) the effect of elite football involvement on sport and academic career success and satisfaction.

Due to a lack of a valid semi-structured tool for the evaluation of women athletes' perceptions and opinions in relation to the quality, challenges, problems, concerns, and needs of their dual career path, a tailored semi-structured electronic tool was developed. In profiting from the co-authors' extensive knowledge and proven experience in the field of elite sport, dual career of sportspersons, and the football community, the research team engaged in focus groups to develop the tailored instrument to be used in the present study. Firstly, a pool of both closed-ended and open-ended items to be organized into thematic sections has been preliminary generated in relation to the core research objectives. Then, a second focus group has been implemented to: (i) discuss the coverage and relevance of the content and structure of the semi-structured tool; (ii) identify potential needs to reformulate and/or implement questions; (iii) reach a consensus on the structure of the tool (e.g., number of sections; number of items; items' typology), on the clarity of the content and wording of each selected item, and on the grouping of items deemed relevant to collect the necessary information coherently with the scope of the study; and (iv) to define clear procedures for questionnaire administration, data acquisition, and data extraction and analysis with no pre-conceptions and/or interreferences with participants' opinions. Finally, a preliminary version of the semi-structured questionnaire was piloted through internal testing within the research team (72). Hence, a 25-items version of the instrument was considered suitable to be electronically administered for data collection.

To produce a homogenous sample, a criterion-based sampling was adopted considering the following inclusion criteria: (i) being an elite Italian female football player, actively involved in the 2023 national Serie A championship; and (ii) being or having being enrolled in a dual career path over her football career. The exclusion criteria included: (i) having quitted the football career in 2023; and/or (ii) having lowered her competition level in 2023. Potential respondents were preliminarily contacted through social media platforms and direct contact with their Football clubs' managerial staff. Upon the expression of interest to participate in the study, participants were recontacted by email to provide them with further information regarding the aim of the study, their expected involvement upon participation, and the specific features of the semi-structured questionnaire. Participants were ensured that their participation was voluntary, the confidentiality of their responses, the right to refuse to answer specific questions, and the option of dropping out at any time without providing any reason. After having collected a written informed consent, participants were provided with the web link to access the semi-structured questionnaire.

To avoid potential bias in the analytical process of open-ended items responses, two members of the research team engaged in repeated, recursive readings of all open-ended responses to reach a consensus on the identification and revision of the major themes. In case of disagreement in the analytical process of open-ended items responses, a third author's opinion was sought.

### 2.2 Participants

Out of 80 preliminary contacts among Italian professional teams competing in the National Serie A (e.g., Como, Fiorentina, Inter, Juventus, Milan, Parma, Roma, Pomigliano, Sampdoria, Sassuolo), 40 players expressed their interest to participate in the study and received the electronic questionnaire. Among them, 7 subjects (17.5%) did not respond to the questionnaire, whereas 11 (27.5%) responses were considered not suitable for data analysis due to missing data. Conversely, 22 responses (response rate = 55%) were considered eligible for data extraction and analysis. In particular, Italian women elite footballers participating in the present study were aged 25.8 ± 4.3 years (range: 20–33), held different education levels (e.g., High school 59%; Bachelor 18%; Master's Degree 23%), and lived in different Italian macro-regions (e.g., North 41%; Centre 50%; South 9%).

### 2.3 Instrument

Table 1 presents the structure of the tailored 25-item semi-structured questionnaire used in the present study, consisting of four main thematic areas as follow:

• Demographic information (e.g., age, educational attainment, geographical position, competition level; items 1–4);
• Dual Career path information (e.g., difficulties, received support from both sports and academic environments, recognition as student-athlete, access to a formal dual career path; items 5–17);
• Dual career and gender issues (e.g., perceived gender inequalities in dual career for football players; items 18–21);
• Career satisfaction (e.g., perceived satisfaction with the dual career path and with sports and academic achievements; items 21–25).

**Table 1:** Semi-structured questionnaire on dual career and gender equality experiences in Italian football.

Area	Items	Text	Response
Demographic information	Item 1	What is your age (years)?	O-E
	Item 2	What is the highest educational qualification you have held?	C-E (MC)
	Item 3	Where your football club is geographically located?	C-E (MC)
	Item 4	What is your sporting level?	C-E (MC)
Dual career path	Item 5	Did you encounter any difficulties in combining your studies with your football career progression?	C-E (D)
	Item 6	If yes, can you describe the main difficulties?	O-E
	Item 7	In which course of study did you encounter the greatest difficulties (for example, during the school period or during the university period, or both)?	C-E (MC)
	Item 8	Can you elaborate on the previous answer, providing more details regarding the experiences you have had and the critical issues you have encountered?	O-E
	Item 9	During your studies, did your sports club support you in combining your sporting and study commitments?	C-E (D)
	Item 10	If so, can you provide details regarding the received support?	O-E
	Item 11	During your studies, did the coaches and technical staff with whom you worked in your football career support you in combining your sporting and study commitments?	C-E (D)
	Item 12	If so, can you provide details regarding the received support?	O-E
	Item 13	During your studies, did the school and/or university where you studied support you in combining your sporting and study commitments?	C-E (D)
	Item 14	In combining your football career with your school/university education, have you been able to benefit from the recognition of your student-athlete status?	C-E (D)
	Item 15	If so, were you able to access a formally recognized dual career program?	C-E (D)
	Item 16	If so, can you describe what type of school/academic support was provided to you?	O-E
	Item 17	If not, can you describe why you were not granted with the student athlete status?	O-E
Dual career and gender issues	Item 18	In your opinion, is it more difficult for women in football to access dual career programs than their men counterparts?	C-E (S)
	Item 19	Can you elaborate on the previous answer with a comment?	O-E
	Item 20	In your opinion, is it more difficult for women in football to be a student-athlete than their men counterparts?	C-E (S)
	Item 21	Can you elaborate on the previous answer with a comment?	O-E
Career satisfaction	Item 22	Are you satisfied with how you were able to combine your school/academic career with your football career (dual career)?	C-E (S)
	Item 23	How satisfied are you with your football career and sporting achievements?	C-E (S)
	Item 24	How satisfied are you with your school/academic career and achievements?	C-E (S)
	Item 25	Can you elaborate on the previous answers?	O-E

C-E, closed-ended; O-E, open-ended; MC, multiple choice; D, dichotomous; S, Likert scale 1–6 pt., where lower scores indicate low agreement/satisfaction, and higher scores indicate high agreement/satisfaction.

In general, closed-ended items aimed at collecting quantitative information regarding major research themes considering different response typologies (e.g., scale, dichotomous, and multiple choice), whereas open-ended items were designed to allow participants deepening their responses by providing comments, details, and additional information.

### 2.4 Data analysis

Collected responses were aggregated based on data typology (e.g., closed-ended and open-ended items). Based on closed-ended item typologies (e.g., multiple choice; dichotomous; scale), quantitative analysis encompassed frequency of occurrence (*n*; percentages, %) and mean and standard deviation calculations (score computation). For qualitative analysis of open-ended items responses (73), inductive (e.g., players' personal experiences) and deductive (e.g., conceptual framework of potential gender biases in accessing and progressing a dual career path in Italian elite football) data analyses were used. In particular, six phases characterized the thematic analysis: familiarization, coding, theme development, refinement, naming, and writing up (74). Then, individual raw quotes were coded and categorized into sub-themes, to be expressed as frequencies of occurrence (e.g., number of times each sub-theme was cited within collected open comments). Multiple readings of the text, the involvement of two authors in the data analysis, and the extraction of major themes to be discussed until consensus was reached ensured the minimization of potential bias in the process. Furthermore, in case of disagreement in the analytical process of open-ended items responses, a third author's opinion was sought (73).

## 3 Results

Results relative to both closed-ended and open-ended items responses are presented in Tables 2, 3, respectively. In particular, Table 2 presents quantitative data (e.g., scale items: scores as mean and standard deviation; dichotomous and multiple-choice items: frequency of occurrence, *n*) derived from closed ended items, whereas Table 3 reports the frequency of occurrence (*n*) of cited subthemes in relation to open-ended items. Major findings proving an integrated and comprehensive picture of participants' responses to both closed- and open-ended items will be presented discursively in relation to two macro-themes: (i) dual career experiences; and (ii) gender impact on dual career paths and career satisfaction.

**Table 2:** Frequency of occurrence and scores of responses to closed-ended items.

Dual career experiences (Item 5, 9, 11, 13, 14, 15)	Yes (*n*)	No (*n*)
Difficulties in combining sport and education (item 5)	13	9
Support from football club received (item 9)	13	9
Support from coaching staff received (item 11)	13	9
Support from school/university received (item 13)	8	14
Student-athlete status recognition (item 14)	7	15
Formal dual career path provision (item 15)	5	2
Gender influence on dual career paths and career satisfaction (item 18, 20, 22, 23, 24)	Score (pt) Mean SD	
Greater difficulties for women players to access dual career paths (item 18)	4.5	1.5
Greater difficulties for women players to be a student-athlete (item 20)	3.7	1.5
Dual career satisfaction (item 22)	3.9	1.5
Football career satisfaction (item 23)	4.7	1.2
Academic career satisfaction (item 24)	4.0	1.2
Which path was more difficult (item 7):	(*n*)	
High school	10	
University	10	
Both	2	

22/22 (100%) respondents.

**Table 3:** Frequency of occurrence of major themes emerged from responses to open-ended items.

Item and topic	Respondents (out of 22 participants)	Cited themes	(*n*)^a^
Item 6: Issues in DC paths	*n* = 14 (64%)		
		Lack of time for class attendance and individual study	5
		Lack of support from teaching staff	3
		Migration issues (mobility for football, but not for university)	3
		Mobility (long travels) to take university exams	3
		Football and academic schedule issues	3
		Lack of formal opportunities and support for DC	2
		Lack of rest time	1
		Proximity issues (distance between the university and the football facility)	1
		Lack of future prospects in the football career	1
		Lack of recognition of the professional status for Serie B players	1
		No issues encountered (good time management is needed)	1
		No issues encountered (training sessions scheduled in the evening)	1
Item 8: Difficulties in managing DC paths	*n* = 14 (64%)	Cited themes	(*n*)
		Lack of flexibility for absences and class/evaluation schedule, also due to travels for games	8
		Lack of rest time and need for good time management	5
		Lack of time for study between school hours and training sessions	3
		Lack of understanding from teaching staff	1
		Proximity issues (distance between the university and the football facility)	1
Item 10 and 12: DC support (sports environment)	*n* = 20 (46%)	Cited themes	(*n*)
		Flexibility for training absences in case of exams and or intensive periods of study	9
		Flexibility in training schedule	9
		Academic tutor provision	1
		Academic career prioritized over the football career	1
		Sensibility from coaches (of the stress deriving from a DC path)	1
Item 16: DC support (academic environment)	*n* = 5^b^ (100%)	Cited themes	(*n*)
		Flexibility in the academic schedule (university) for class attendance and exams	4
		Flexibility in school evaluations	3
		Justified absences for class attendance	3
		Recognition of training sessions as working internship	1
		Academic tutor	1
Item 17: Lack of student athlete status recognition	*n* = 14 (82%)	Cited themes	(*n*)
		Lack of recognition as a student-athlete in the past	4
		Gender difference in the eligible competitive level to be considered a student-athlete (me*n* = Serie C; women = Serie B)	4
		Administrative issues derived from the start of a new dual career national programme (lack of consistent procedures and practices)	3
		I was not aware of DC opportunities	2
		Lack of recognition within the working environment	1

Item 19: Gender-related inequalities in accessing DC paths	*n* = 13 (59%)	Cited themes	(*n*)
		Lack of development policies in the past for women's football, leading to a lack of recognition for women student-athletes	4
		Gender equality in football is a hot topic (as in other social domains) and an ongoing process	3
		Men's football attracts more interest, leading to more opportunities for male student-athletes (i.e., private academies, agreements with schools/universities)	3
		Belief that women's football is less demanding than that of men's, leading to a lack of resources and support	2
		A DC path means that athletes need to study because they do not earn enough money from their football career	1
		Lack of financial resources and time for managing a DC	1
Item 21: Difficulties for women to be a student-athlete	*n* = 13 (59%)	Cited themes	(*n*)
		Pay gap (women tend to dedicate more to their study path due to limited income from football; conversely, men have higher salaries, leading to a lower need to invest in their academic career)	8
		Football related demands are less recognized for women (i.e., a hobby)	4
		Men in football have more opportunities	2
		Lack of time for managing a DC	1
		There are no gender differences	1
Item 25: Career satisfaction	*n* = 10 (45%)	Cited themes	(*n*)
		The football career was negatively influenced by the academic career (relocation decisions, no past professional recognition)	4
		The academic career was negatively influenced by the football career (time management)	2
		Satisfied with the academic career, but could have done more	2
		Satisfied with the football career, but willing to do more	2
		Not satisfied of a study path in an online university	1

DC, dual career.

^a^ (*n*) = frequency of occurrence (*n*) of citations received by each subtheme within collected open comments.

^b^ Item 16: out of 5 respondents involved in a formal dual career path.

### 3.1 Dual career experiences

Regarding dual career experiences, 60% of participants encountered difficulties in their dual career path (item 5), independently from the education level (around 50% for both secondary and tertiary education; item 7). Among the major difficulties affecting the combination of their elite sport and academic demands, 64% of participants reported the lack of time for class attendance and individual study, lack of support from teachers/professors and academic institutions, migration issues (e.g., mobility for pursuing better football career conditions, but constraining their academic path), mobility to undertake university exams, and football and academic schedule issues. In particular, the players highlighted:

“In high school, I had little time to study and the teachers never sustained my dual career efforts (the contrary!). During my university path, the only problem was having to move for football reasons (vocational mobility), while not having the opportunity to change the University”

“Making long trips to take exams because the university was far from the field where I played was challenging, and with the university not meeting the needs of the athlete”.

“During school I lived at home and played near home. During my university years I started to travel for football as a work, and it was difficult to organize timetables and transfers to the university, making it impossible to attend classes”.

Furthermore, 64% of respondents highlighted (item 8) time management issues due to the lack of flexibility for absences and for class/evaluation schedule, the lack of rest time, and the lack of time for study between school hours and training sessions as a major factor affecting their dual career, as reported in the following comments:

“Time management has always been an issue in my dual career. In Italy, compared to other countries, no merit is recognized for people who carry out an excellent academic and athletic career, making the combination of sport and academic demands almost incompatible”.

“Absences for class attendance were never justified. Lack of availability of educational institutions to activate educational paths with alternative timetables in terms of frontal lessons and/or exams”.

Regarding supportive measures, most of the respondents (74%) highlighted a support received from the sport environment (item 9 and 11), mainly in the form of flexibility for training absences in case of exams and or intensive study periods, and flexibility for training schedule (item 10 and 12; 46%). Conversely, educational institutions were generally perceived as less supportive (36%; item 13), as reported in the following comments:

“Despite my extra-curricular commitment at both Serie A and national level, I was always treated by the teachers as if my only daily commitment was school”.

“Coaches and football club supported me by accommodating my training schedule, giving me more time to study, and the possibility of having instructors who helped me with my studies.”

“Making themselves (coaches) available for my study plan, as much as possible”.

When this aspect was further investigated (item14), the student-athlete status during the dual career path was recognized only for 7 participants (32%), with only few (28%) of them benefiting of accessing a formal dual career path (item15) mostly consisting in flexibility in the academic schedule, class attendance and exams/evaluations, and justified absences (item 16). Conversely, 82% of the sample with no recognized student-athlete status and/or formal access to a dual career programme in the past reported the lack of awareness towards student-athletes' needs, gender differences relative to the eligible competitive level to be considered a student-athlete (e.g., men; Serie C-third league; women: Serie B-second league), and administrative issues as dual career constraining factors (item 17). In particular, one player shared a relevant experience in this regard:

“During my school and academic years most of the female footballers could not apply for dual career services and the student-athlete recognition. In both school and university, all football competitive levels were recognized for male colleagues, whereas only Serie A was the minimum eligible level for women players. At the university, I contacted the Equal Opportunities Committee to report this inequality. After various conversations with the managers of the application for dual career services, the same competitive levels were recognized for both male and female players.”

### 3.2 Gender influence on dual career paths and career satisfaction

Regarding the perceived gender influence on dual career paths, participants reported higher difficulties in accessing a dual career programme for women players (item 18). As major reasons substantiating the gendered patterns of dual careers in football, 59% of participants reported: the lack of development policies in the past for women's football (leading to a lack of recognition for women as student-athletes); the infancy stage of the gender equality discourse within the field of football; the higher interest around men's football, thus attracting more economic resources and interests, leading to more opportunities for male football student-athletes (i.e., private academies, agreements with educational institutions); and the belief that women's football is less demanding than that of men's, leading to a lack of resources and support.

However, gender was not perceived as a constrain for being a student-athlete dedicated to combine effectively sport and academics (item 20). In particular (item 21), 59% of participants reported that women tend to dedicate more to their educational path due to lower financial resources derived from their football career, whereas men footballers were perceived to have a lower need to invest in their academic career in light of high football-related incomes, and more opportunities to access a dual career programme. Furthermore, the lower recognition of football-related demands of women football considered more as a hobby was also highlighted, as reported in the following comments:

“Women's football in Italy is only recently gaining recognition from a social and professional point of view. It follows that the recognition of the professional athlete status for female footballers is still a very hot and controversial topic today, and for this reason it is still the subject of debate and struggle for the recognition of equal rights with respect to male colleagues. And not just in the world of football.”

“Football for women is not compared to men’s football as there are enormous differences in multiple areas (infrastructures, financial resources, etc.), this provides a distorted perception to the outside world (women football as less demanding).”

“The perception of women who play a male-dominated sport leads to thinking that for women it is just a hobby and not a real commitment”.

Regarding the perceived career satisfaction, dual career paths (item 22) received the lowest score (3.9 ± 1.5 pt.), whereas intermediate and highest scores emerged for specific academic attainment (item 24; 4.0 ± 1.2 pt.) and football achievements (item 23; 4.7 ± 1.2 pt.), reflecting participants' commitment to succeed in both the athletic and educational spheres. Although respondents reported a general satisfaction with their career outcomes, further insights (45%) were provided in responses to item 25, highlighting the mutual constrain determined by sport and academic demands in absence of a formal dual career programme, as highlighted in the following comments:

“Having a daily football commitment that takes up the whole morning leads to having less time for studying. I am happy for having fulfilled my university obligations for this year (exams) and I am also aware that with more time I could often have obtained higher grades.”

“Combining my football career with my academic was really difficult for me, without the help of the University and schools in general I always had to organize myself as best I could, trying to reconcile everything. It wasn't always easy, and both school and sporting results have been affected.”

“I feel satisfied with the results I managed to obtain at university level, with the little time I was able to dedicate to them. I am not fully satisfied on my football achievements because I believe that if I had had the opportunity to follow my university career more easily, I would have been able to experience football at a higher level, and eventually to profit of higher national (and maybe international) football mobility.”

## 4 Discussion

The main objective of the present study was to investigate gender and dual career intersecting inequalities in Italian elite football by means of the views of women elite footballers, and to explore difficulties in combining sport and academic commitments that might be overcome through the recent professional recognition of women's football, which might facilitate their career development in a male-dominated industry. The main findings generally confirmed previous literature in the area of gender in football (27, 30–33, 35–39, 56, 75, 76), with women having struggled to be recognized as professional elite athletes, and receiving lower welfare and support measures for their career development with respect to their men footballer counterparts, including their dual career.

### 4.1 Women's football and dual career support from an institutional perspective

Although male and female players dedicate similar efforts to achieve sporting success, participants highlighted that women's commitment to football is still perceived as a hobby rather than a professional involvement in elite sport and/or a tangible career opportunity, with women's football perceived as less demanding and not requiring particular efforts to accommodate sports, academic, working, and life commitments (27, 35–40). The present study highlights the issues female footballers faced in the past years to be recognized as elite players and to successfully pursue a dual career, as well as the lack of services that might have facilitated their career path. In this framework, the recent recognition of the full-time status would have a positive impact in the future development of women's football at national level, as well as conditions for a dual career in Italian female players.

A positive outcome from the present study is the support participants received from the football environment (e.g., club, coaching staff), mainly in the form of flexibility for training absences in case of exams and or intensive periods of study and in training schedule. Although previous literature on dual career of student-athletes at sport level reported a general lack of flexibility in adaptation of training schedule (77, 78), the present findings attributed a relevant dual career role to coaches who care the holistic development of the athletes supporting their commitment in formal education, thus challenging the culturally dominant discourse that flexibility should pertain mainly the educational institutions (20, 49, 53). In fact, the participants in the present study reported a lower support received from academic institutions, questioning the opportunity, efficacy, and availability of academic services for student-athletes. In light of the full-time recognition to elite women footballers, the improvement of dual career services offered by educational institutions should be envisaged.

In absence of dual career support services and with limited financial resources and time availability, athletes might experience high levels of stress leading to sport or academic drop-outs, thus limiting the possibility to pursue a professional sport career or delaying education to pursue high athletic achievements (79, 80). In this study, the players' comments highlighted the gender differences in accessing dual career opportunities, which were due to lack of development policies for women's football and the consequent lack of resources and support services. Coherently, the negative impact of one career over the other was highlighted. Interestingly, one participant emphasized that “involving in a dual career path means that athletes need to study because they do not earn enough money from their football career”, confirming previous research showing that women athletes tend to prioritize their academic career to increase their future career prospects (57–59, 81, 82). In fact, being the gender pay gap substantial, a low opportunity to capitalize for the life course and uncertain financial future push female players towards a higher involvement and dedication towards their education compared to their male counterparts (35–37, 80). In this framework, a disconnection between education and football was confirmed, with investments in academic achievements seen as a wise plan when foreseeing post-sport career transitions in the labor market (57). Although participants in this study reported a good overall satisfaction in relation to their career achievements and outcomes, their low satisfaction for dual career at academic level urges measures to achieve a successful combination of football and educational demands, which would support the athletes in time management skills for developing multiple areas of their life (19, 83–85).

### 4.2 Dual career issues and support in relation to gender

Specific to their dual career experiences, respondents reported difficulties well documented in previous research (9–11, 13, 14, 17, 18, 20, 86), especially within academic environments (e.g., flexibility in class attendance, lack of time for individual study, lack of support from teaching staff). Women players also reported that migration and national relocation to pursue better football career outcomes determined issues in combining their football demands with the academic career, mainly due to mobility (e.g., lack of proximity between sports facilities and educational institutions, long travels to take university exams) and time schedule constrains. Migration and/or relocation and/or mobility burdens also affected the necessary rest time and the private and social life of athletes, which represents a relevant component of a well-balanced dual career path (9, 20, 77, 87, 88). This finding should be considered also in light of the lack of both formal opportunities and support for dual career in women's football, requiring them to place high efforts to succeed in sport and academics, as stated in previous research in the field (35–37, 80). Whilst Italian educational institutions offered dual career programs to male players competing already at the third league (Serie C) level or included in elite youth football academies, only sub-elite (Serie B) and elite (Serie A) women players were recognized to be eligible for the student-athlete status, as substantiated by the only five participants reporting to have been enrolled in a formal dual career program adjusting their academic schedules. In considering that a dual career impact elite athletes' life at multiple (e.g., sporting, academic, psychological, socio-cultural, and financial) levels (10, 11, 13, 15, 19, 89) and the high economic impact of football (25), more resources should be allocated to implement dual career support services (e.g., psychological support, counselling, tutoring, financial support) to elite and talented players. Furthermore, the football community at large should show a higher awareness towards the recognition of the student athletes' needs, independently from their gender. In compliance with international (3–8) and sports-specific (41) recommendations and quest for a gender mainstreaming, concrete actions should be taken at national and local levels not only to increase the number of women football players but also to ensure them equal dual career opportunities and conditions. The perceived gender discrepancy has been substantiated also by two respondents reporting no knowledge of dual career opportunities determined by a lack of information provided through the academic and sports institutional communication channels and/or sensibility towards the needs of elite female student-athletes. Thus, it would seem appropriate to further invest to properly disseminate available opportunities and services in both sports and educational organizations for elite student-athletes (20, 47, 90).

## 5 Conclusions and future directions

The present study contributed to shed light on the presence of gender issues in the pursue of a dual career in the Italian football context, with women facing additional challenges to combine their sport and academic demands with respect to their men counterparts, especially due to the lack of recognition, support, and available services. Although the present findings could not be considered conclusive in providing a comprehensive picture on the state of art of dual career support in the European elite football context, it represents the first attempt to collect preliminary information on perceived intersecting gender and dual career inequalities in Italian football through the involvement of elite women football players. In considering the declared intention of FIGC to implement several policy actions in women's football (43) (e.g., to increase the number of registered young female players by 50% by 2025, to achieve international successes, to improve the competitiveness and spectacle of the competitions, and to increase the fan base), the introduction of professionalism for women players in 2022 into Serie A represents the first step towards the achievement of the strategic goals. Future studies should be envisioned to deepen the understanding and knowledge regarding women elite players' career development and trajectories, with a particular focus on the effects of professionalism on their sport and education choices, also in relation to potential differences with respect to their men counterparts.

Whilst ensuring the sustainability of the championship at sporting level might represent *a priority*, in implementing strategies towards gender equality in football FIGC should consider also other aspects, such as fair working conditions, equal pay, student-athlete recognition, and dual career services. To date, although the recent recognition of women's professional status should be considered a milestone towards equality in this sport discipline, discrimination and abuse of female players has not been completely overcome (91). Surely, a comprehensive approach would challenge stereotypes, dismantle barriers to equal recognition of men's and women's football, and advocate for the necessary change towards a more inclusive and equitable future for all athletes, regardless of gender. Thus, synergic, multistage, and multidimensional cooperation with all the involved stakeholders is necessary to implement strategies fostering a more inclusive and supportive environment for female athletes in football in the future through the addressing of general and specific issues and challenges derived from the lack of gender mainstreaming in the past.

Future multistage and multidimensional policy implementation should be envisioned, taking into account evidence-based information regarding barriers, opportunities, needs, and necessary support for successful dual career paths, also in relation to fair and equal conditions for both female and male players. In this regard, this approach would not only nurture a stronger link between scientific research and evidence and real implementation, but also address specific challenges and necessary actions based on a solid priority framework. At specific sport policy level, FIGC should continue along the necessary multi-stage journey at national level to further align with FIFA and UEFA policies towards gender equality in football. This should include multiple short-, mid-, and long-term actions, to be planned and implemented at different levels, encompassing the enlargement of female participants in the sport, the allocation of fundings for grassroots development, further investments on women's leagues at different competition levels (e.g., junior and senior; local, regional, national and international competition levels), the fostering of the professionalization and financial sustainability of the whole women's football sector, the challenging of the pay gap through the synergistic involvement of elite football multiple stakeholders (e.g., leagues, clubs, sponsors, players' associations, media), the promotion of women in coaching, refereeing, and executive roles, and the establishment of stronger anti-discrimination, -harassment, and -abuse policies. Coherently, at local, regional and national levels football organizations should also comply with international and national policies through the design and implementation of programs aligned with women's football strategic objectives (e.g., girls and women football courses, investments in women's teams, visibility of women's championships, agreements with educational stakeholders to promote dual careers). Finally, a higher awareness in the not-sporting community and stakeholders at large (e.g., parents of athletes, educational institutions, teachers/professors, dual career service providers) should be envisioned to facilitate dual career footballers in their career path and to equip them with the necessary skills to cope with the difficulties occurring during their career transitions. Hence, educational bodies and personnel (e.g., governmental; schools and universities; teaching and administrative personnel) should be involved in this process, promoting the dialogue and cooperation with sporting organizations and the reinforcement of dual career services for elite footballers, independently of their gender.

The current study provides novel insights into the women players' perceptions on gender equality and dual career welfare in the Italian elite football context. However, the present findings must be considered in light of several limitations. Firstly, the limited sample size challenges the generalizability of findings. Indeed, although our sample might be considered representative of women football players competing in elite Italian football, a higher number of participants should be envisioned in future studies to provide a more comprehensive picture of female players' challenges and needs in relation to their career development, trajectory, and dual career-related issues. Secondly, having the study the aim to highlight perceived gender disparities in elite Italian dual career footballers, a sample composed by only women challenged the possibility to gain further insights from reported diverse career pathways and experiences in relation to gender. Thus, future research should envision the inclusion of both male and female players to allow a direct comparison and to shed light on different gendered perceptions on elite football and dual career pathways within the Italian context. Thirdly, a data collection made through an electronic semi-structured questionnaire could have limited the depth of the collected information, since self-reported data could introduce response and recall biases. Thus, triangulating and mixed-method study designs (e.g., integration of questionnaire responses, in-depth interviews, and/or institutional data) should be considered in future research.

To conclude, to explore the different perceptions on career opportunities and development, and to identify possible strategies to promote gender mainstreaming in football, further studies are needed enlarging the sample size, involving male and female players as well as various football and dual career stakeholders, and applying mixed-methods approaches for data collection. Furthermore, both national and cross-national research in this area should be considered to allow comparisons and to identify potential best practices to be exploited within and beyond national contexts to a broader community devoted to the promotion of gender mainstreaming in football careers.

## Data Availability

The raw data supporting the conclusions of this article will be made available by the authors, without undue reservation.
